# On the Hydrolytic Depolymerization of Polyurethane Foam Wastes by Ionic Liquids

**DOI:** 10.3390/molecules30173523

**Published:** 2025-08-28

**Authors:** Rebeca Salas, Rocio Villa, Francisco Velasco, Maria Macia, Virtudes Navarro, Jairton Dupont, Eduardo Garcia-Verdugo, Pedro Lozano

**Affiliations:** 1 Departamento de Bioquímica y Biología Molecular B e Inmunología, Facultad de Química, Universidad de Murcia, Campus de Espinardo, E-30100 Murcia, Spain; r.salasvidal@um.es (R.S.); francisco.velascod@um.es (F.V.); j.dupont@um.es (J.D.); 2 Departamento de Química Inorgánica y Orgánica, Universidad Jaume I, Campus del Riu Sec, E-12071 Castellon, Spain; maciam@uji.es (M.M.); cepeda@uji.es (E.G.-V.); 3 Centro Tecnológico del Mueble y la Madera, Región de Murcia, C/ Perales S/N, E-30510 Yecla, Spain; v.navarro@cetem.es

**Keywords:** polyurethane foam waste, chemical recycling, ionic liquids, recycled polyols, sustainability

## Abstract

Flexible polyurethane foams (PUFs) are widely used materials whose crosslinked chemical structure hinders conventional recycling, leading to significant environmental challenges. This study presents a selective and scalable depolymerization strategy for polyurethane foam waste (PUFW), utilizing a combination of 1-butyl-3-methylimidazolium chloride ([Bmim][Cl]) as water-miscible ionic liquid (IL) and a strong organic base to enable hydrolytic cleavage of urethane bonds under mild reaction conditions (98 °C, atmospheric pressure). The approach was evaluated across different PUFW formulations and successfully scaled up to a 1 kg reaction mass, maintaining high efficiency in both the depolymerization and separation steps. The recovered polyols exhibited high purity and structural fidelity, comparable to those of virgin polyols. The recycled products were integrated into a new foam formulation, resulting in a PUF with mechanical and morphological properties, as revelated by scanning electron microscopy (SEM), which closely resemble those of virgin polyol-based references and surpass those of foams produced using commercially recycled polyols. These findings support the feasibility of closed-loop polyurethane recycling and represent the transition towards circular polymer economy strategies.

## 1. Introduction

The increasing urgency of transitioning towards more sustainable industrial models, particularly since the adoption of the United Nations Sustainable Development Goals (SDGs) in 2015, has placed significant pressure on the polymer industry to reduce environmental impact and embrace the circular economy principle [1,2,3,4,5]. Within this context, green chemistry provides a fitting framework for designing products and processes that minimize waste, improve resource efficiency, and reduce dependence on fossil-based inputs [6,7,8,9].

Among synthetic polymers, polyurethane (PU) holds particular significance due to its widespread use and end-of-life management challenges. PU is produced via polyaddition reaction between polyols and di- or poly-isocyanates in the presence of an organic or organometallic catalyst, resulting in a crosslinked network of urethane bonds (–NH–CO–O–). Both the crosslinking degree and the nature of the soft and hard segments in the polymer matrix determine its mechanical and thermal properties. Among PU-based products, flexible polyurethane foams (PUFs) are especially challenging due to their low density, diverse molecular architecture and thermoset nature, which greatly complicates their storage and recycling [10]. Unlike thermoplastics, which consist of linear or branched chains and can be melted, the three-dimensional covalent bonding in PU prevents remelting and reshaping. This structural rigidity is a key factor that contributes to PUF’s chemical resistance and recycling challenges, particularly in post-consumer waste streams [11].

Since their invention in 1937 by Dr. Otto Bayer [12], PUs have become ubiquitous in consumer and industrial applications such as mattresses, furniture, packaging, and automotive interiors. Nearly 90 years later, PUs rank as the sixth most produced polymer globally, with over 24 million tons manufactured annually [13]. In Europe, more than 170 industrial plants produce PU foam. However, despite their widespread use, PU materials present a significant environmental burden at end-of-life due to their limited recyclability, increasing landfill costs, and tightening environmental regulations. In fact, most PUF waste is disposed of via landfilling or incineration, potentially damaging the environment [14,15].

These factors collectively underscore the urgent need for developing more sustainable PU waste management strategies, with a particularly focus on recycling methodologies. Among them, chemical depolymerization strategies enable the recovery of valuable monomers, particularly polyols, which can be reused in the production of new PU materials [16]. Compared to other strategies based on biocatalytic depolymerization [17,18], which remains largely at the exploratory stages, chemical methods have advanced further towards industrial implementation [19,20,21]. These approaches fall into two main categories: recovery of isocyanates and recovery of polyols. For instance, O’Dea et al. demonstrated an approach for depolymerizing PUs at 80 °C in toluene, using a Lewis acid organoboron catalyst (β-chlorocatecholborane), which enabled the recovery of isocyanate monomers for the production of second-generation PU materials [22].

However, the dominant industrial focus has been on polyol recovery, as it offers a more direct pathway to material circularity. Given that polyol content typically accounts for 60–70% of the total foam mass, its full recovery represents a notable gain in recyclability and resource efficiency. Thus, recovered polyols (RPs) can replace virgin polyols (VPs) in new PU formulations, significantly reducing reliance on petroleum-based feedstocks. Conventional depolymerization strategies, such as glycolysis and hydrolysis, typically involve harsh conditions, including high temperatures (150–280 °C), long reaction times (6–48 h), and the need for energy-intensive solvent removal steps (e.g., vacuum distillation of glycol excess). These processes are often inefficient and generate dark-colored polyols, primarily due to uncontrolled side reactions, which reduce their quality and industrial value [23,24,25,26].

Indeed, previous studies have shown that the incorporation of chemically recycled polyols can lead to variations in foam properties, depending on purity, recycling method, and substitution level. For example, Kraitape et al. reported that increasing recycled polyol content could alter cell morphology and reduce shape-recovery at higher loadings, although tensile and compressive strengths improved [27]. Other studies have found that small additions (1–5 wt%) of microwave-assisted glycolysis polyol decreased hardness but enhanced mechanical strength, particularly in polyester-based foams [26]. Similarly, it has been demonstrated that polyols obtained via microwave-assisted aminolysis can yield foams with properties comparable to virgin references [28]. Collectively, these findings highlight that high-purity recycled polyols can maintain or even improve performance, reinforcing the relevance of developing selective depolymerization methods for high-quality RP production.

However, the current mismatch between PU production and recycling capacity continues to grow. While PU manufacturing has matured into a large-scale global industry, recycling technologies remain underdeveloped. However, companies like the Rampf Group^®^ (Allmendingen, Germany) [29] and H&S Anlagentechnik^®^ (Sulingen, Germany) [30] are actively researching PU recycling strategies, underscoring the significance of this field. Notably, RAMPF technology has already been in industrial use at the Recpur^®^ plant (Puertollano, Spain) since 2024 [31]. Although this is a modest beginning after nearly nine decades of continuous PU production, bridging this technological and temporal gap is essential for closing the loop in the PU lifecycle.

**Figure 1:**
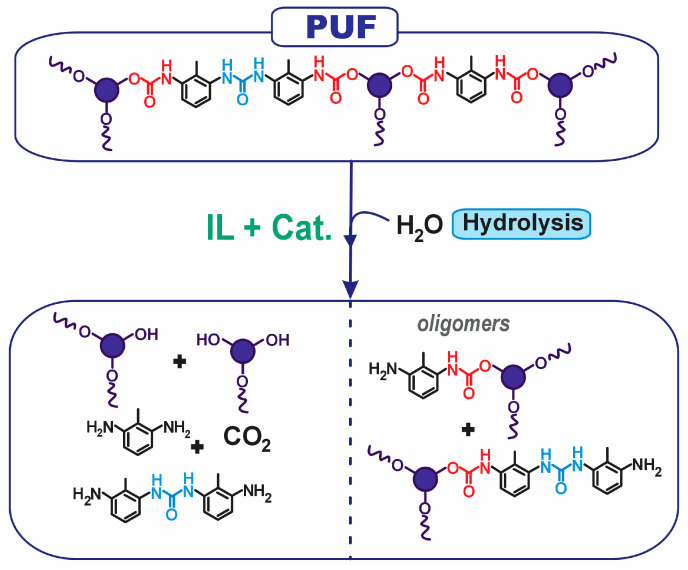
General scheme of the hydrolytic depolymerization of PUFW. The top section illustrates the representative chemical structure of a typical PUF, featuring urethane (highlighted in red) and urea (in blue) linkages. The bottom section shows the principal hydrolytic products.

It should be noted that ionic liquids (ILs) have emerged as non-innocent solvents that demonstrate potential in polymer chemistry for both syntheses, depolymerization and recycling [32]. In previous work, a sustainable depolymerization strategy for PUFWs based on the synergistic action of ionic liquids (ILs) and cyclic amidine-derived superbases has been developed. This system enables selective cleavage of urethane bonds under mild conditions (<100 °C), realizing valuable RPs [33,34]. The approach was designed to align with the principles of green chemistry, offering both robustness and potential for industrial scalability. The present study aims to evaluate the industrial viability of the hydrolytic depolymerization of PUFWs enabled by IL technologies and basic catalysts to recover valuable RPs (Figure 1). This challenge will be addressed by means of three key aspects: (i) assessing the versatility of the system across different PUFWs, (ii) demonstrating its scalability, and (iii) validating the reusability of the RPs in the synthesis of new PUFs.

## 2. Results and Discussion

The present approach is based on the combination of [Bmim][Cl] as a water-miscible IL medium combined with a strong organic base with a minimum pKa (in water) above 13 (e.g., 1,8-diazabicyclo[5.4.0]undec-7-ene [DBU], pka = 13.5), establishing an active depolymerization medium (DM) able to catalyze the hydrolytic cleavage of the urethane bonds present in the foam structure. As illustrated in Figure 1, the depolymerization products include (i) RPs, (ii) CO_2_, (iii) aromatic amines and their oligomeric derivatives (e.g., 2,4-/2,6-toluenediamine (TDA) fragments), and (iv) oligomeric species retaining intact urethane moieties. These products reflect the cleavage efficiency and mechanistic selectivity of the depolymerization system under mild reaction conditions.

**Figure 2:**
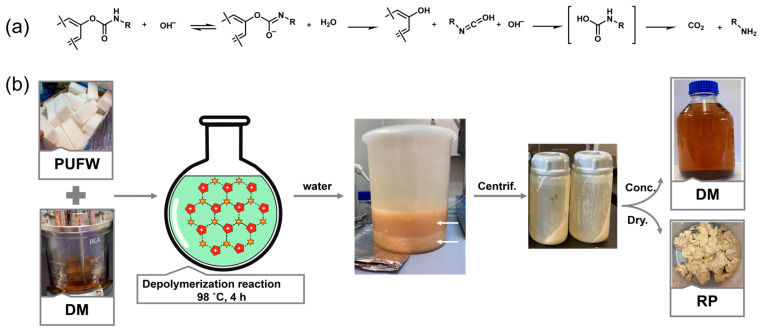
(**a**) Mechanism of base-induced hydrolysis of carbamates. Adapted from ref. [36]. (**b**) Workflow of the depolymerization and recovery process for PUFW. From left to right: combination of the PUFW and the DM ([Bmim][Cl], DBU, and H_2_O) at 98 °C for 4 h. After reaction time, the subsequent water addition and centrifugation step induce phase separation: a solid phase consisting of RP and an upper aqueous phase containing the DM. Final images show the clean isolation of the RP as a white solid and the DM as a clear amber liquid.

While the detailed mechanism remains under investigation, previous studies suggest a base-activated nucleophilic hydrolysis pathway (see Figure 2) [35,36]. In this process, the strong base deprotonates water, generating hydroxide ions that selectively attack the electrophilic carbonyl carbon of the urethane moiety, leading to efficient bond cleavage. The IL facilitates this reaction not only by solubilizing the crosslinked PU matrix but also by stabilizing charged intermediates through hydrogen bonding and ionic interactions [37,38,39,40]. This dual function exemplifies the behavior of a “non-innocent solvent,” where the solvent actively modulates the reaction pathway beyond its conventional role as a medium [41].

Furthermore, an easy and efficient protocol was established for the recovery of both the RP and the DM. The addition of water facilitates the precipitation of the polyol—along with possible oligomeric species retaining urethane moieties—as a white, insoluble solid. Meanwhile, the IL and base catalyst remain fully dissolved in the aqueous phase, which can be readily separated and reused. Overall, this process represents a closed-loop chemical recycling strategy with strong potential for industrial application (Figure 2).

### 2.1. IL and Superbase Catalyst-Driven Hydrolytic Depolymerization of Different PUFWs

**Figure 3:**
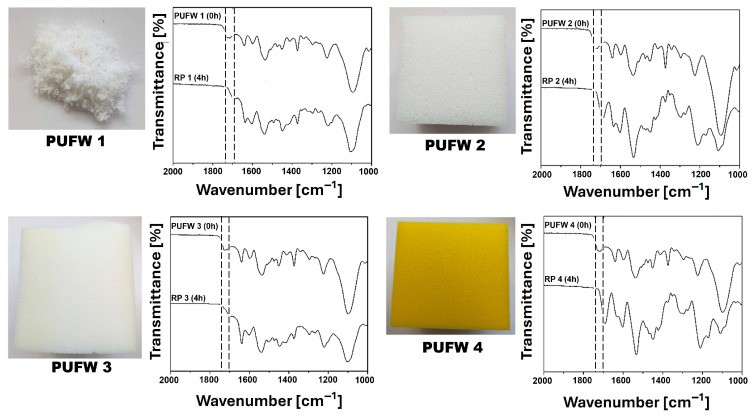
Comparative analysis of the FTIR spectra of the original PUFWs (0 h) and the RPs (4 h) obtained after the DBU-catalyzed hydrolysis depolymerization of different PUFWs in [Bmim][Cl] (4 h, 95 °C). The characteristic band of the urethane bond (1700–1740 cm^−1^) is indicated by a dashed line.

To assess the versatility and practical applicability of the depolymerization system, flexible polyether-based PUFWs were used due to their widespread industrial use in bedding, seating, and packaging applications, as well as their higher hydrolytic stability compared to polyester-based analogues [42]. Consequently, the performance of this approach was evaluated across several PUFWs with distinct formulations: PUFWs 1, 2, and 3 were synthesized from toluene diisocyanate (TDI 80/20; 29 wt%) and a single polyol component (Alcupol F-4811- Repsol, Puertollano, Spain; 67 wt%), with minor differences in additives (e.g., catalysts and surfactants). In contrast, PUFW 4 was prepared from a mixed polyol formulation comprising Alcupol P-3041 and Alcupol F-4811 in a 9:1 weight ratio (67 wt% total), combined with TDI (29 wt%) (see Figure 3). These differences are representative of common industrial formulations and provide a relevant framework for evaluating the robustness of the depolymerization strategy.

In all cases, depolymerization reaction was conducted using a fixed reaction medium composed of [Bmim][Cl]:DBU:H_2_O (62:13:25 wt%), with a PUFW loading of 25 wt% (1 g). After 4 h at 95 °C, the reaction mixtures were cooled at room temperature, and the addition of water led to the formation of two perfectly clear phases: (i) a solid phase corresponding to the RP, and (ii) aqueous fraction containing both the IL and superbase catalyst. The resulting solid products were then isolated, washed, and dried.

Subsequently, the RPs were analyzed by Fourier-transform infrared (FTIR) spectroscopy to confirm the structural integrity of the product. The complete disappearance of the characteristic urethane carbonyl absorption band at ~1720 cm^−1^ across all the recovered solids confirmed the cleavage of urethane linkages (Figure 3).

These results highlight the broad substrate tolerance of the technology, demonstrating its effectiveness and robustness across industrially relevant PUF formulations, an essential feature for real-world recycling applications.

### 2.2. Evaluation of the Scale-Up Performance and Efficiency of PUFW Depolymerization

Once the efficiency and selectivity of the IL–superbase depolymerization strategy has been demonstrated, the next key step was to evaluate its scalability and possible potential applicability. In this context, it should be noted that industrial implementation requires that both the depolymerization reaction and downstream separation processes remain stable over time, while also robust and efficient at larger scales. Accordingly, two scale-up experiments were conducted to assess both the operational feasibility of the catalytic system and its recovery performance. In both cases, the catalytic medium consisted of the previously described system: [Bmim][Cl]:DBU:H_2_O at a weight ratio of 62:13:25%.

**Figure 4:**
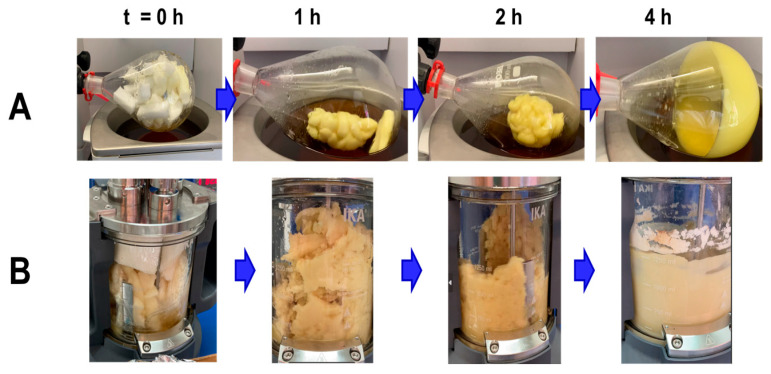
Visual progress of the scale-up depolymerization reactions of (**A**) 20 g of PUFW executed in a rotary evaporator setup, and (**B**) 200 g of PUFW conducted in an IKA LR 1000 basic reactor over 4 h at 98 °C.

Firstly, the mass of PUFWs was increased by 20 times compared to the previous experiments, and the depolymerization reaction was carried out in a rotary evaporator equipped with a 2 L round-bottom flask and a thermostatically controlled glycerol bath (see Figure S1). The mixture was then stirred by rotation and maintained at 98 °C for 4 h. Complete solubilization and depolymerization of the foam were achieved under these conditions (Figure 4A).

Alternatively, the second trial was carried out in an IKA LR 1000 basic reactor (see Figure S2) to depolymerize up to 200 g of PUFW in a total reaction mass of 1 kg, representing a 200-fold increase from the initial assays. Once again, complete breakdown and homogeneous dispersion of the foam were observed within 4 h at 98 °C (Figure 4B), demonstrating that the system maintains its efficiency across a two-order-of-magnitude scale increase.

Scaling up PU depolymerization approaches involves challenges that exceed the straightforward adjustment of initial experiment setups to higher volumes, especially because of the low density, high porosity, and compressibility of flexible foams [43]. These physical properties hinder mixing and mass transfer, especially in standard stirred-tank reactors. Therefore, efficient mechanical agitation is essential in overcoming these limitations. During the second experiment, foam fragmentation was visually apparent within the first hour, suggesting enhanced kinetics due to improved reactor stirring. This effect likely arises from increased impeller torque and higher fluid inertia, which facilitated better dispersion of PUF within the viscous IL-reaction medium. Particularly, PUF systems demand careful optimization of hydrodynamic parameters to ensure reaction efficiency [44,45,46,47].

The downstream product separation protocol, which is based on water addition, phase splitting, and centrifugation, was also evaluated at these larger scales. Figure 2 shows the complete depolymerization process, from raw materials through to product isolation. As mentioned above, following the depolymerization reaction, the addition of water resulted in RP precipitation as a white solid, while the DM remained in the aqueous phase, confirming straightforward and efficient separation.

To evaluate the efficiency of the depolymerization process at larger scales, a mass balance was performed for both the RP and the DM (see Table 1). Notably, the second experiment conducted at a larger scale (200 g PUFW) exhibited a higher polyol recovery (90.6%) compared to the first assay (81.5%), indicating improved process performance. The observed slight mass losses were attributed to the release of CO_2_ as gaseous byproducts, the possible presence of trace amounts of free amines soluble in the aqueous phase, and operational handling steps, which are known to cause slight but cumulative losses during processes. Remarkably, in both scaled-up experiments, more than 98% (by weight) of the IL:catalyst system was successfully recovered, underscoring its potential for recyclability and demonstrating the robustness of the system for industrial implementation.

Overall, these results confirm that the present approach is both chemically efficient and operationally scalable up to a total reaction mass of 1 kg, corresponding, in our largest trial, to 200 g of PUFW processed with 500 g [Bmim][Cl], 100 mL DBU, and 200 mL water. The method not only preserves chemical selectivity and reaction efficiency at larger volumes, but also enables the straightforward recovery of high-purity RP and DM. Hence, the technology is positioned at Technology Readiness Level (TRL) 3–4, representing a validated laboratory-scale proof of concept [48]. After successful validation of the process at larger laboratory-scale, the next step was to investigate the performance of the recovered polyols in the formulation of new PUFs under representative industrial conditions.

### 2.3. Characterization of the Recycled Polyol

Prior to direct reintegration of the obtained RP into new PUF production, it is essential to confirm its suitability by assessing its physicochemical properties. Accordingly, the RP obtained from the scaled-up experiments was thoroughly analyzed and characterized using various analytical methods.

A key initial observation was that the RP obtained from the present technology appeared as a bright white solid RP, in contrast to the colorless liquid form of the VP (Alcupol F-4811, Repsol, Puertollano, Spain) (Figure S3). This contrasts sharply with the darkened products typically observed in other depolymerization methods, which operate at elevated temperatures (≥180 °C), and frequently generate side-products via uncontrolled thermal degradation and oxidation [26,49,50]. This striking appearance suggests a more selective and controlled depolymerization reaction, notably devoid of thermal degradation by-products such as aromatic amines.

Among the most concerning degradation by-products in such processes are the carcinogenic monomeric aromatic diamines, such as TDA, which impair product quality and hinder reuse [51]. In contrast, the mild operational conditions of the proposed process (≤98 °C) significantly suppress such side reactions. Detailed analysis by ^1^H-NMR (Figures S9 and S10) confirms that the RP consists predominantly of high-purity polyol, with only minor amounts of aromatic oligomers. Free aromatic amines, such as TDA, were detected only in very low concentrations. Specifically, the RP, isolated as a white solid, exhibited minimal aromatic signals, corresponding to a quantified amine concentration of 0.47 mM, while the DM contained 0.768 mM of free aromatic amines (see Supporting Materials for more details). Overall, these results confirm both the chemical selectivity of the depolymerization system and the efficiency of the downstream separation protocol.

**Figure 5:**
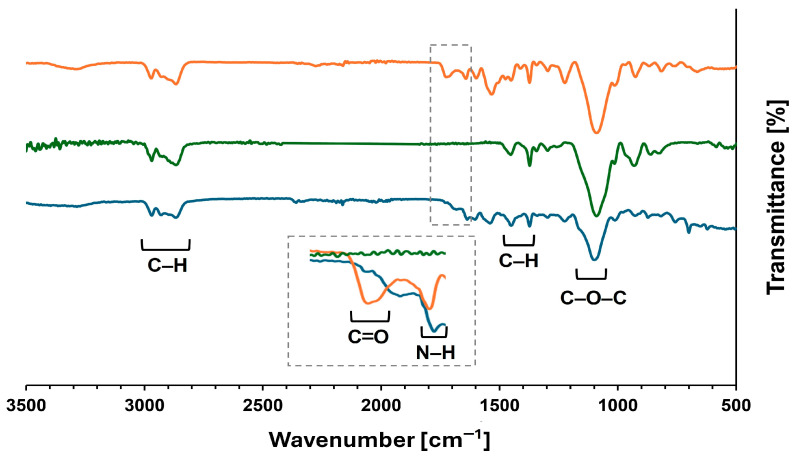
Comparative analysis of the FTIR spectra of the original PUFW (orange) and VP (green) with the RPs (blue) obtained after the DBU-catalyzed hydrolysis reaction in [Bmim][Cl] (4 h, 98 °C). Dotted lines refer to the region wherein relevant vibrational bands of carbamate and urea groups are present.

To confirm the structural fidelity of the recovered product, a comparative FTIR analysis was performed on the PUFW, VP, and RP (Figure 5). All samples exhibited characteristic polyol absorption bands: C–H stretching vibrations in the 2800–3000 cm^−1^ region, C–H bending at 1448 cm^−1^ and 1375 cm^−1^, and a strong C–O–C stretching band at 1094 cm^−1^ associated with polyether segments [49]. Furthermore, the retention of key polyether spectral features, specifically the C–O–C stretching and alkyl C–H bending bands, indicates that the polyether backbone remained intact.

Crucially, the PUFW displayed a distinct absorption band at ~1720 cm^−1^, corresponding to the urethane C=O stretch, which was significantly reduced in the RP spectrum. This confirms that the urethane bonds were successfully cleaved under the reaction conditions assayed. Trace residual intensity at this wavenumber may be attributed to the presence of oligomeric fragments containing intact urethane linkages.

Further characterization of the RP included the determination of its hydroxyl number value, measured at 52.5 mg KOH/g, closely matching that of the VP (45.4 mg KOH/g). This small deviation remains within acceptable flexible foam formulation limits [52]. In addition, the RP exhibited full solubility in DMSO, equivalent with the solubility behavior of its virgin counterpart. This full solubility in DMSO (>99%) corresponds to the overall depolymerization conversion, as it refers to the depolymerization of the main substrate (PUFW), which is insoluble in organic solvents, unlike the recycled product obtained.

Altogether, these findings validate the high quality of the RP in terms of purity, structure, and reactivity. Its white appearance and essential physical parameters indicate that the IL–superbase depolymerization strategy not only meets technical specifications but also addresses key industrial and regulatory concerns. This underscores the method’s potential as a sustainable route for PUF recycling.

### 2.4. Production of New PUF Using Recycled Polyols

After confirming the efficiency, selectivity, and scalability of the depolymerization strategy, the next step was to evaluate the industrial applicability of the RP by reintegrating it into the preparation of a new flexible PUF. This assessment was carried out in collaboration with Interplasp S.L., a manufacturer with previous expertise in producing commercial PUFs using recycled feedstocks [53].

To benchmark the performance of the obtained RPs, comparative foam synthesis experiments were carried out using three types of polyols (see Table 2):

1. A virgin flexible polyether polyol (VP; Alcupol F-4811, Repsol);
2. A commercially available recycled polyol (CRP; Repsol Reciclex^®^, Repsol, Puertollano, Spain);
3. The recovered polyol produced through the process described in this work (RP).

Prior to its use, a blend of RP and VP was formulated at a 1:1 weight ratio (see Figure S3), since it was necessary to have a liquid solution for the foam synthesis reaction to be possible.

**Figure 6:**
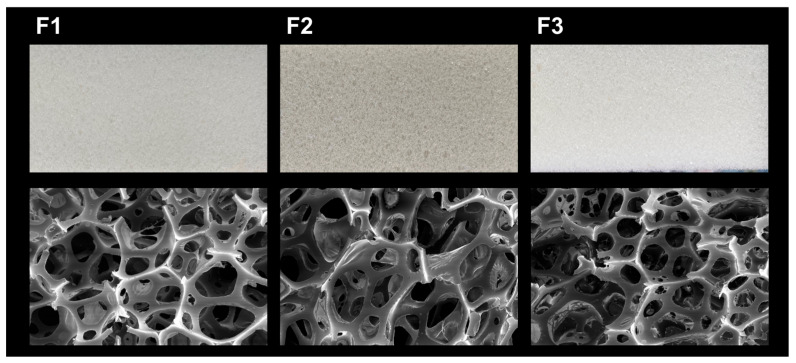
Photographs and scanning electron micrographs of cross-sections of the three PUFs synthesized according to the formulation in Table 2. Foam samples include (**F1**) reference foam prepared with 100 wt% VP, (**F2**) foam incorporating 5 wt% of a commercial recycled polyol (CRP; Repsol Reciclex^®^), and (**F3**) foam containing 5 wt% of the recycled polyol (RP) obtained via the IL–superbase hydrolysis method.

Two flexible foams were synthesized using 5 wt% of recycled polyols in the formulation, along with a reference foam prepared exclusively with VP. All three foams expanded properly, achieving comparable volumes and open-cell morphology (see Figure 6). The most immediate visual distinction was observed in coloration: the foam synthesized using CRP (F2) showed a brown tint, commonly associated with residual aromatic amines such as TDA. In contrast, the RP-based foam (F3) was bright white, visually indistinguishable from the virgin reference foam (F1). This consistency is desirable in many industrial applications, particularly those where product appearance is decisive.

Scanning electron microscopy (SEM) allowed for a cell structure assessment, confirming that all foams exhibited a typical open-cell structure with spheroidal cells, uniform size distribution, and interconnected pores, all desired features for flexible PUFs [54,55]. These results were further validated by a broader survey of multiple foam sections, all of which showed consistent cellular features (see Figure S11). Furthermore, a quantitative measurement of the cell diameters in the foams using representative images was performed. It showed diameters of 273 µm for the virgin-polyol control foam (F1) and 275 µm for the foam prepared from recycled polyol (F3), indicating that the incorporation of RP did not measurably affect cell size.

In contrast, the foam produced from commercial recycled polyol (CRP, F2) displayed slightly larger cells, with an average diameter of 305 µm, potentially reflecting less uniform curing or structural heterogeneity in the feedstock (see Figure S12). Larger cell sizes in PUF have been linked to decreased uniformity and can negatively affect mechanical performance, such as reducing compressive strength, particularly when foam density remains constant [56]. Importantly, no structural defects (e.g., cell collapse, voids, etc.) were observed in any foam, underscoring that the obtained RP supports robust cellular integrity.

Additionally, Table 2 shows the influence of the substitution of VP with recycled polyol on the physical properties of flexible PUFs. All foams exhibited similar rise times, though F3 was more similar to F1 than to F2. The foam density of the reference foam was 26.57 kg/m^3^, very similar to the values of the CRP and RP foam (26.45 kg/m^3^ and 26.08 kg/m^3^, respectively). Notably, the slight density decrease observed for F3 (~0.5 kg m^−3^ lower than F1) could represent an economic advantage in applications where foam is sold per unit volume, as less material mass would be required for the same volume. Hardness measurements showed that RP produced a slightly softer foam (2.88 kPa/s) compared to F1 (2.94 kPa/s), whereas CRP yielded a higher hardness (3.20 kPa/s).

Foam porosity is the parameter that indicates how many openings on the surface of the material are present within the material. Foams that are high in pores are more permeable, which means they will allow more liquids and gases to pass through them, hence affecting other parameters like foam airflow (the parameter that indicates the close cell versus open cell content of the polymer). Flexible polyurethane foams are characterized by a lower level of close cell content, allowing for a good breathability [57]. The results in Table 2 point out that the recycled polyol base foam slightly decreased the porosity of the material, whilst maintaining values very close to the reference (65 and 66% vs. 68%). These values suggest that the RP maintains the high open-cell content required for breathability, while minor differences are unlikely to affect end-use performance. Overall, the close agreement in morphology, density, hardness, and porosity between RP-based and virgin-based foams demonstrates that the polyol recovered via the present depolymerization method is functionally equivalent to virgin polyol in flexible foam applications.

Recent studies have shown that it is possible to substitute significantly higher percentages of virgin materials with their recycled counterparts in flexible PUFs without substantially degrading performance [58]. Indeed, life cycle assessment (LCA) analyses, which examine the process from cradle to grave, have further demonstrated that foams formulated with high RP content yield superior environmental profiles across several impact categories, provided that functional performance is maintained [59].

Taken together, these results highlight the feasibility of incorporating RP into flexible PUFs; however, formulation adjustments may be necessary when targeting higher substitution ratios to ensure consistent mechanical and processing behavior. In this context, the ability to selectively recover and reuse polyols within today’s industrial framework provides a practical and scalable step towards circular PU manufacturing. Building on these results, future work will focus on increasing the percentage of RP in commercial foam formulations, with the goal of fully replacing fossil-derived polyols in selected applications.

## 3. Materials and Methods

This section outlines the reagents, materials, and experimental protocols used for the development, execution and validation of the PUFW depolymerization strategy.

### 3.1. Reagents

The reactants used in this study included the ionic liquid 1-butyl-3-methylimidazolium chloride ([Bmim][Cl], 99% purity, CAS No. 79917-90-1), purchased from IoLiTec-Ionic Liquids Technologies GmbH (Heilbronn, Germany), and the basic catalyst 1,8-diazabicyclo[5.4.0]undec-7-ene (DBU, 98% purity, CAS No. 6674-22-2), from Sigma-Aldrich (Hamburg, Germany).

Additional reagents included dimethyl sulfoxide (DMSO) from Carlo Erba Reagents S.A.S. (Barcelona, Spain), pyridine from Scharlab S.L. (Barcelona, Spain), and deuterated DMSO (DMSO-d_6_), phthalic anhydride, sodium hydroxide (NaOH), all from Sigma-Aldrich (Hamburg, Germany).

All other chemicals were purchased from Sigma-Aldrich and were used in the highest available purity.

### 3.2. Flexible Polyether Polyurethane Foam Waste

Four different flexible polyether PUFW samples were used in this study. All were supplied by the company Interplasp S.L., based in Yecla (Murcia, Spain).

Foam synthesis was performed under standard industrial conditions via condensation between toluene diisocyanate (TDI 80/20, CAS No. 584-84-9; 29 wt%) and polyol components (67 wt%) as main raw materials. The polyol phase consisted either of pure Alcupol F-4811 (used in PUFW 1, PUFW 2, and PUFW 3) or a 9:1 wt/wt mixture of Alcupol P-3041 and Alcupol F-4811 (used in PUFW 4).

The synthesis reaction occurred at ambient temperature (22.2 °C), atmospheric pressure (944 mbar), and 20% relative humidity at 2200 rpm mixing speed. The formulation included Tegostab BF2370 (stabilizer), Tegoamin B75 (amine catalyst), and Kosmos T9 (tin octoate catalyst) (see Table 3).

### 3.3. Depolymerization of the Polyurethane Foam Waste

Depolymerization tests were performed on all available PUFW samples using a model reaction. Glass vials (26 mL) were charged with [Bmim][Cl] (2 g, 11.45 mmol), water (0.6 mL, 33.3 mmol), and DBU (0.4 mL, 2.85 mmol). Then, 1 g of PUFW was added to each vial. The mixtures were stirred at 95 °C for 4 h under constant agitation.

### 3.4. Separation Protocol for Recycled Polyol and Depolymerization Medium

To efficiently recover the depolymerization products (RP and DM), an optimized separation protocol was used. After the reaction time, deionized water (twice the volume of the original reaction mixture) was added, leading to two distinct phases: (i) a lower precipitated solid phase containing the recycled polyol, and (ii) an upper aqueous phase containing the IL and catalyst in solution.

The two phases were separated by centrifugation at 6000 rpm for 10 min at room temperature. The resulting solid (RP) was washed and centrifuged twice with fresh water to improve separation and purity. Afterwards, it was dried to constant weight in an oven and stored for further analysis or reuse. The liquid phase (water/IL/catalyst) was concentrated under reduced pressure (100 mbar, 45 °C) to recover the MD for subsequent cycles.

### 3.5. Depolymerization Scale-Up Experiments

For the first experiment, a depolymerization medium was prepared by mixing [Bmim][Cl] (50 g, 2.86 mol), DBU (10 mL, 0.71 mol), and water (20 mL, 11.1 mol). Then, 20 g of PUFW 3 were added, and the mixture was stirred at 98 °C for 4 h at 100 rpm in a rotavapor evaporator set up. After the reaction, the recovery protocol described before was applied.

For the second experiment, a depolymerization medium was prepared by mixing [Bmim][Cl] (500 g, 2.86 mol), DBU (100 mL, 0.71 mol), and water (200 mL, 11.1 mol). Then, 200 g of PUFW 3 were added, and the mixture was stirred at 98 °C for 4 h at 100 rpm in an IKA LR 1000 Basic. After the reaction, the recovery protocol described before was applied.

### 3.6. Analysis and Characterization

To evaluate the efficiency of the PUFW process, a series of physicochemical analyses were performed on the RPs and DMs. These techniques enabled the confirmation of urethane bond cleavage, assessment of structural integrity, and the validation of the functional suitability of the RPs for reuse in PU formulations.

#### 3.6.1. Fourier-Transform Infrared Spectroscopy (FTIR)

Infrared spectra were recorded using a Jasco FTIR-4700 spectrometer (Madrid, Spain), operating in attenuated total reflectance (ATR) mode over the spectral range of 400–3500 cm^−1^, with a resolution of 4 cm^−1^. Before each measurement, an atmospheric background spectrum was collected (32 scans, 4 cm^−1^ resolution). Solid or liquid samples were placed directly onto the ATR crystal for analysis.

FTIR analysis of the RPs confirmed depolymerization of the PUF materials (complete or partial), as evidenced by the disappearance of the characteristic carbonyl absorption band associated with urethane linkages (–NH–CO–O–), typically observed between 1700 and 1740 cm^−1^.

#### 3.6.2. Hydroxyl Number Determination

The hydroxyl number of the recovered products was determined using the standard titration method described in ASTM D-4274-16 [60]. This assay involves the esterification of hydroxyl groups with phthalic anhydride (0.25 M) dissolved in pyridine at an elevated temperature (98 °C). The excess, unreacted anhydride is then titrated with a standardized sodium hydroxide solution (NaOH, 0.25 M).

For the analysis, accurately weighed samples (~100 mg) of thoroughly dried polyol were placed in individual glass vials with 5 mL of the anhydride solution. A blank reference vial containing only the anhydride solution (without polyol) was used to establish the baseline NaOH consumption.

Each sample was stirred until fully dissolved, then heated at 98 °C for 2 h under constant agitation. After cooling to room temperature, 50 μL of phenolphthalein indicator solution (20 mg/mL in pyridine) was added to each vial. The samples were titrated with 0.25 M NaOH until a persistent pink color appeared for at least 15 s.

(1) $$\mathrm{Hydroxyl}\;\mathrm{Number}\;(\mathrm{mg}\;\mathrm{KOH}/g)=\frac{\left(B-A\right)\cdot C\cdot MW\cdot 1000}{g},$$

The hydroxyl number was calculated using Equation (1), where A is the volume of NaOH consumed for the sample (mL), B is the volume of NaOH consumed for the blank (mL), C is the NaOH concentration (0.25 M), MW is the molecular weight of KOH, and g is the sample mass (in grams).

This procedure allowed precise quantification of the functionalization level of the recycled polyols and their suitability for future application in polyurethane foam formulations.

#### 3.6.3. Solubility Test in Dimethyl Sulfoxide

A solubility test in dimethyl sulfoxide (DMSO) was also performed to assess the effectiveness of the depolymerization process. This test relies on the fact that intact PUFs are insoluble in DMSO due to their high molecular weight and crosslinked structure. However, after cleavage of urethane bonds during depolymerization, the polymeric matrix breaks down into lower-molecular-weight polyol fragments, which are soluble in polar solvents such as DMSO.

Thus, solubility in DMSO serves as a straightforward indicator of successful bond cleavage. Complete dissolution of the solid in DMSO visually confirms the conversion of PU into soluble compounds and provides practical evidence of the chemical process’s efficiency.

For the assay, 50 mg of RP (obtained after precipitation and drying) were dissolved in 2 mL of DMSO. Since intact PU does not dissolve in DMSO, full dissolution of the sample was taken as a positive indicator of effective depolymerization.

#### 3.6.4. Nuclear Magnetic Resonance (NMR) Spectroscopy

Experiments were conducted on a Bruker Avance 400 and 600 MHz spectrometer.

To confirm urethane bond cleavage and to elucidate the structure of the resulting products, NMR spectroscopy was performed on both RP and MD.

For the concentrated DM, 50 μL of the sample were dissolved in 435 μL of DMSO-δ_6_ and 15 μL of *tert*-butanol (1 M) in DMSO-δ_6_.

Alternatively, for the dry solid product (RP), 40 mg were dissolved in 500 μL of DMSO-δ_6_, then centrifugated. Finally, 485 μL of the supernatant were mixed with 15 μL of *tert*-butanol (1 M) in DMSO-δ_6_.

#### 3.6.5. Scanning Electron Microscopy (SEM)

Samples were mounted on aluminum stubs with carbon tape and platinum sputter-coated with a 5.0 nm thin layer (Leica EM ACE 600, Leica Microsystems GmbH, Wetzlar, Germany). FESEM imaging was done at 5 kV with secondary electrons. FESEM-EDX analyses were performed with a FE-SEM (Thermofisher, ApreoS Lovac IML, Waltham, MA 02451, USA,) and coupled with an AMETEK Octane Plus EDAX microanalyzer (Berwyn, PA 19312, USA) at 20 kV.

## 4. Conclusions

This work demonstrates a significant advancement in sustainable PU recycling by developing a mild, efficient, and scalable depolymerization system based on a water-miscible IL and superbase catalysts. The method achieves effective chemical breakdown of PUFWs at temperatures below 100 °C and ambient pressure, yielding high-purity polyols that are easily separated and readily reusable in new PUF formulations. Furthermore, the straightforward product recovery, where polyol precipitates upon water addition, allows the recovery of white, high-purity polyols, enhancing commercial value and aesthetics.

Depolymerization was successfully applied to multiple flexible PUFW samples, highlighting the method’s robustness and operational flexibility. In large-scale trials (up to 1 kg batch mixture, ~200 g PUFW), the obtained RP was thoroughly characterized and subsequently employed in the formulation of new PUFs under realistic industrial conditions. The resulting RP-based foams exhibited an average cell size (275 µm), density (26.08 kg m^−3^), hardness (2.88 kPa), and porosity (66%) essentially equivalent to the virgin-polyol control (273 µm, 26.57 kg m^−3^, 2.94 kPa s, 68%, respectively), while avoiding structural defects such as cell collapse or void formation. In contrast, foams prepared from a commercial recycled polyol showed larger cell sizes and minor deviations in hardness and porosity, highlighting the superior quality of the RP obtained via this method.

These results demonstrate that partial substitution of virgin polyol with RP—without compromising mechanical performance or morphology—is feasible and can deliver material quality aligned with industrial specifications. The process offers scalability, operational simplicity, and potential economic benefits from lower-density foams. Future work will focus on increasing recycled content in formulations, adapting the method for more complex PU waste streams, and performing full-scale economic and life-cycle assessments. Overall, this approach provides a practical and high-quality pathway for closing the loop in the PUF lifecycle, contributing directly to circular economy and sustainability targets.

## Figures and Tables

**Table 1:** Mass balance of the scale-up depolymerization trials of PUFW using an IL–superbase hydrolysis system (98 °C, 4 h, [Bmim][Cl]:DBU:H_2_O, 62:13:25 wt%).

Component	Input Mass (g)	Recovered Mass (g)	Recovery (%) ^1^
(20 g PUFW)			
RP fraction ^2^	—	16.3	81.5
DM fraction ^3^	60	59.1	98.5
(200 g PUFW)			
RP fraction	—	181.2	90.6
DM fraction ^2^	600	597.3	99.6

^1^ Recovery percentages are calculated based on the initial input mass; ^2^ Recycled Polyol obtained after precipitation, separation, and drying; ^3^ Depolymerization medium (IL and base catalyst) recovered after aqueous separation and water concentration.

**Table 2:** The influence of the substitution of virgin polyol (VP) with recycled polyol on the properties of flexible PUFs.

	F1	F2	F3
Formulation Changes vs. Table 3			
VP (pphp ^1^)	100.00	95.00	95.00
CRP ^2^ (pphp)	0.00	5.00	0.00
RP ^3^ (pphp)	0.00	0.00	5.00
Foam Physical Properties			
Color	White	Light brown	White
Rise time (s)	137	143	130
Density (kg/m^3^)	26.57	26.45	26.08
Hardness (Kpa/s)	2.94	3.2	2.88
Porosity (%)	68	65	66

^1^ pphp: parts per hundred of polyol; ^2^ CRP: commercially available recycled polyol from Repsol (Repsol Reciclex^®^); ^3^ RP: recovered polyol obtained through the process described in this work.

**Table 3:** Formulation of the polyether foam used in this study.

Foam Formulation	pbw ^a^
VP ^b^	67.00–62.00
RP ^c^	0.00–5.00
TDI 80/20	29.00
Water	2.33
Tegostab BF2370	1.40
Tegoamin B75	0.10
Kosmos T9	0.165

^a^ pbw: parts by weight (used in industrial PU formulations); ^b^ VP: Virgin Polyol; ^c^ RP: Recycled Polyol.

## Data Availability

The original contributions presented in this study are included in the article/supplementary material. Further inquiries can be directed to the corresponding author.

## Supplementary Materials

The following supporting information can be downloaded at https://www.mdpi.com/article/10.3390/molecules30173523/s1, Figure S1. Schematic representation of the rotary evaporator setup used for the scale-up depolymerization experiment. Key components are labeled as follows: (1) Condenser with refrigerated coil, (2) Receiving Flask, (3) Evaporating Flask (2 L), (4) Thermostatic Bath (Glycerol Bath), and (5) Motor Unit.; Figure S2. Schematic representation of the IKA LR 1000 Basic reactor setup used for scale-up depolymerization experiments. Key components are labeled as follows: (1) Heating Jacket, (2) Reactor Vessel, (3) Stirrer Shaft and Impeller, (4) Temperature Probe, (5) Reactor Lid, and (6) Support Handle; Figure S3. Photographs of (A) Virgin Polyol (VP; Alcupol F-4811, Repsol), (B) Recovered Polyol (RP) obtained via the depolymerization method described in this work, and (C) VP:RP blend formulated at a 1:1 weight ratio (w/w; Figure S4. ^1^H-NMR spectra of the VP (Alcupol F-4811, Repsol). The peak assignment refers to the structures in Table S1; Figure S5. ^1^H-NMR spectra of DBU. The peak assignment refers to the structures in Table S1; Figure S6. ^1^H-NMR spectra of [Bmim][Cl]. The peak assignment refers to the structures in Table S1; Figure S7. ^1^H-NMR spectra of 2,4-TDA. The peak assignment refers to the structures in Table S1; Figure S8. ^1^H-NMR spectra of 2,6-TDA. The peak assignment refers to the structures in Table S1, Figure S9. ^1^H-NMR spectra of the depolymerization medium(DM) after concentration, with a zoomed-in view of the 5.7–6.7 ppm region. The peak assignment refers to the structures in Table S1; Figure S10. ^1^H-NMR spectra of the recovered polyol (RP) after concentration, with a zoomed-in view of the 5.7–6.7 ppm region. The peak assignment refers to the structures in Table S1; Figure S11. Scanning electron micrographs of cross-sections of the PUFs synthesized using the formulation described in Table 2, shown at two magnifications (50× and 100×). Foam samples include: (F1) reference foam prepared with 100 wt% VP, (F2) foam incorporating 5 wt% of CRP, and (F3) foam containing 5 wt% of the RP; Figure S12. Scanning electron micrographs of cross-sections of the PUFs synthesized using the formulation described in Table 2, with cell diameters indicated within the images. Foam samples include: (F1) reference foam prepared with 100 wt% VP, (F2) foam incorporating 5 wt% of CRP, and (F3) foam containing 5 wt% of the RP; Table S1. Assignment of characteristic signals in ^1^H NMR spectra; Table S2. Monomeric aromatic amines concentration within the depolymerization medium (DM); Table S3. Monomeric aromatic amines concentration within the recycled polyol (RP).

## Abbreviations

The following abbreviations are used in this manuscript:

[Bmim][Cl]	1-Butyl-3-methylimidazolium Chloride
DBU	1,8-Diazabicyclo[5.4.0]undec-7-ene
DM	Depolymerization Medium
FTIR	Fourier-Transform Infrared Spectroscopy
ILs	Ionic Liquids
NMR	Nuclear Magnetic Resonance
PU	Polyurethane
PUFs	Polyurethane Foams
PUFW	Polyurethane Foam Waste
RPs	Recovered Polyols
SEM	Scanning Electron Microscopy
SDGs	Sustainable Development Goals
TDA	2,4-/2,6-Toluenediamine
TDI	Toluene Diisocyanate
TRL	Technology Readiness Level
VPs	Virgin Polyols
