# Supplementary material for: On the Hydrolytic Depolymerization of Polyurethane Foam Wastes by Ionic Liquids
Source: Molecules. 2025 Aug 28;30(17):3523. doi: 10.3390/molecules30173523 (PMC12430318; doi:10.3390/molecules30173523)
Supplement: Supplementary file 1 [file molecules-30-03523-s001.zip › molecules-3814432-supplementary.pdf]

Supplementary materials

On the hydrolytic depolymerization of polyurethane foam wastes by ionic liquids technology

- **Rebeca Salas[1], Rocio Villa [1,*], Francisco Velasco [1], Maria Marcia [2], Virtudes Navarro [3], Jairton Dupont[1], Eduardo Garcia-Verdugo [2], Pedro Lozano [1,*]**

1 Departamento de Bioquímica y Biología Molecular B e Inmunología, Facultad de Química, Universidad de Murcia. Campus de Espinardo, E-30100, Murcia, Spain.

2 Departamento de Química Inorgánica y Orgánica, Universidad Jaume I. Campus del Riu Sec, E-12071-Castellon, Spain.

3 Centro Tecnológico del Mueble y la Madera de la Región de Murcia. C/ Perales S/N, E-30510, Yecla, Spain.

* Correspondence: plozanor@um.es ; rocio.villa@um.es

Table of contents

1. Supporting Figures and Tables
2. Free aromatic amines quantification
   2.1. Quantification of free aromatic amines in the depolymerization medium.
   2.2. Quantification of free aromatic amines in the recovered polyol.

1. Supporting Figures and Tables

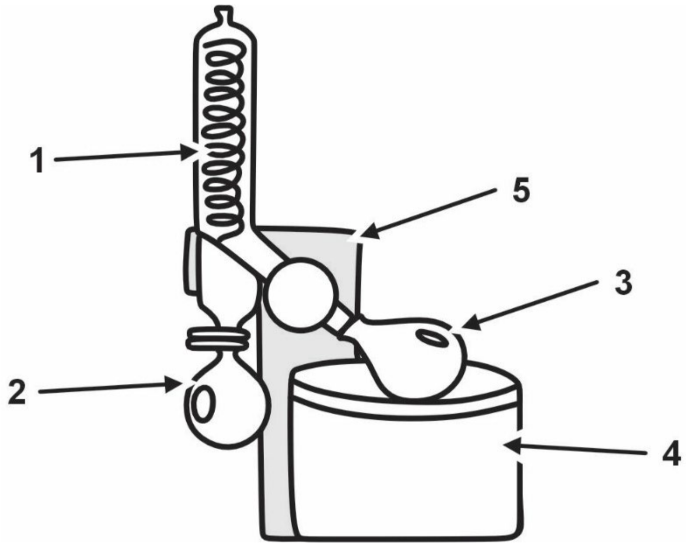

Figure S1. Schematic representation of the rotary evaporator setup used for the scale-up depolymerization experiment. Key components are labeled as follows: **(1)** Condenser with refrigerated coil, **(2)** Receiving Flask, **(3)** Evaporating Flask (2 L), **(4)** Thermostatic Bath (Glycerol Bath), and **(5)** Motor Unit.

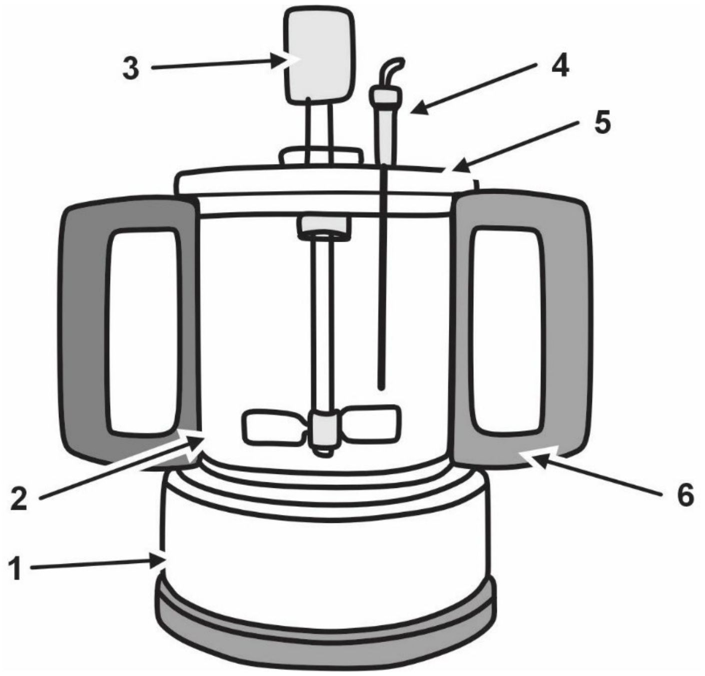

Figure S2. Schematic representation of the IKA LR 1000 Basic reactor setup used for scale-up depolymerization experiments. Key components are labeled as follows: **(1)** Heating Jacket, **(2)** Reactor Vessel, **(3)** Stirrer Shaft and Impeller, **(4)** Temperature Probe, **(5)** Reactor Lid, and **(6)** Support Handles.

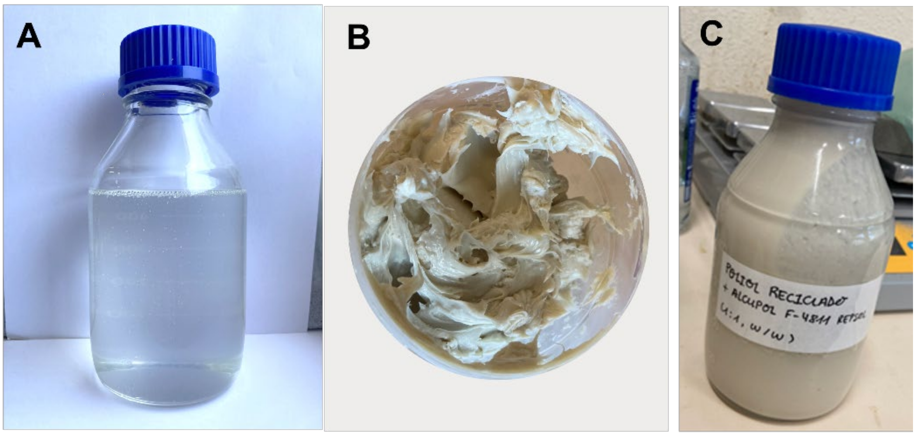

Figure S3. Photographs of **(A)** Virgin Polyol (VP; Alcupol F-4811, Repsol), **(B)** Recovered Polyol (RP) obtained *via* the depolymerization method described in this work, and **(C)** VP:RP blend formulated at a 1:1 weight ratio (w/w).

Table S1. Assignment of characteristic signals in ^{1}H NMR spectra.

Compound	Molecular Structure	Characteristic Chemical Shift(s), ppm[1]
VP: Alcupol F-4811	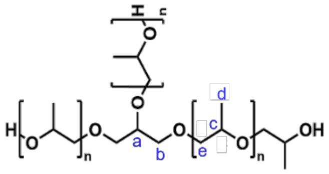	**a, b, c, e**: 3.15-3.70 ppm (m, 6H)<br>**d**: 1.05 ppm (t, 3H)
1,8-Diazabicyclo[5.4.0]undec-7-ene (DBU)	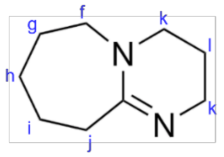	**f**: 3.06 ppm (t, 2H);<br>**g, h, i**: 1.52 ppm (m, 6H);<br>**j**: 2.24 ppm (t, 2H);<br>**k**: 3.15 ppm (t, 4H);<br>**l**: 1.65 ppm (q, 2H)
1-Butyl-3-methylimidazolium chloride ([Bmim] [Cl])	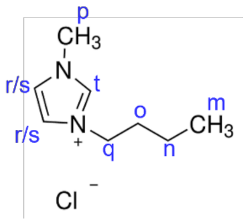	**m**: 0.88 ppm (t, 3H);<br>**n**: 1.24 ppm (sx, 2H);<br>**p**: 1.76 ppm (q, 2H);<br>**p**: 3.87 ppm (s, 3H);<br>**q**: 4.19 ppm (t, 2H);<br>**r**: 7.79 ppm (d, 1H);<br>**s**: 7.87 ppm (d, 1H);<br>**t**: 9.5 ppm (s, 1H)
2,4-toluene diamine (2,4-TDA)	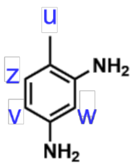	**u**: 2.09 ppm (s, 3H);<br>**v**: 5.77 ppm (d, 1H);<br>**w**: 5.89 ppm (s, 1H);<br>**z**: 6.56 ppm (d, 1H)
2,6-toluene diamine (2,6-TDA)	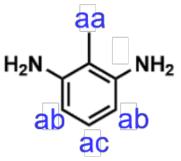	**aa**: 2.09 ppm (s, 3H)<br>**ab**: 5.94 ppm (d, 2H)<br>**ac**: 6.57 ppm (t, 1H)

[1] The abbreviations used are: s (singlet), d (doublet), t (triplet), q (quintuplet), sx (sextuplet), and m (multiplet).

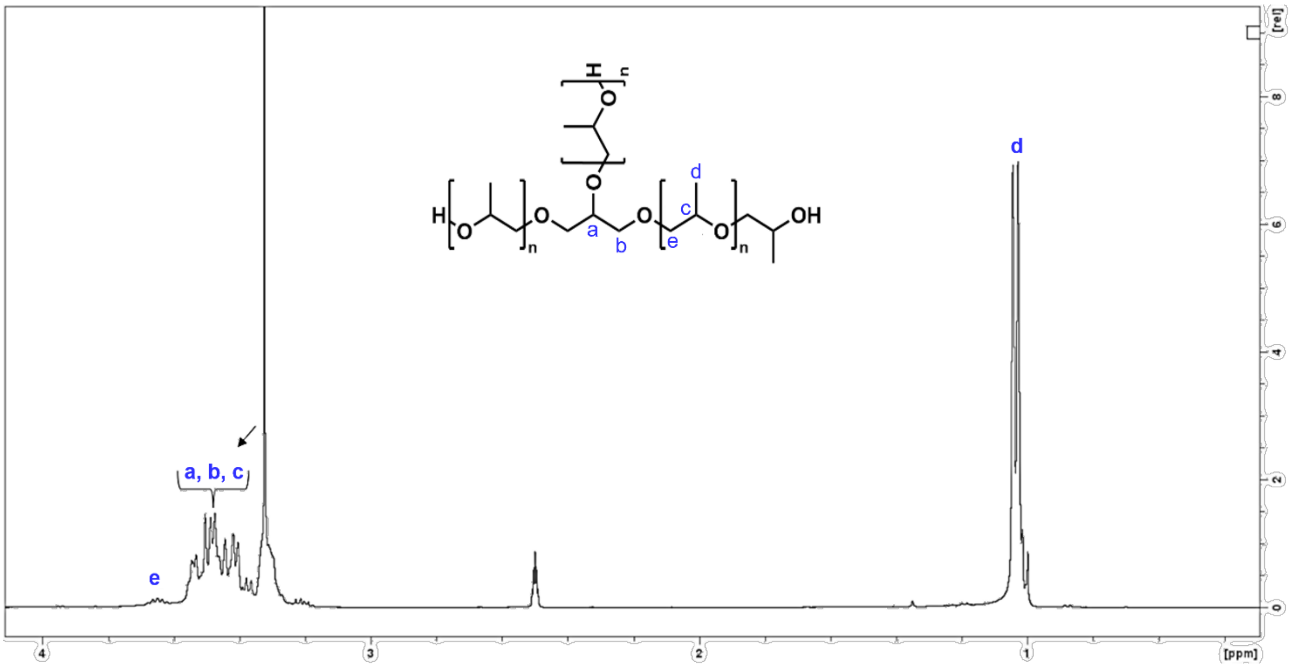

Figure S4. ^{1}H-NMR spectra of the VP (Alcupol F-4811, Repsol). The peak assignment refers to the structures in Table S1.

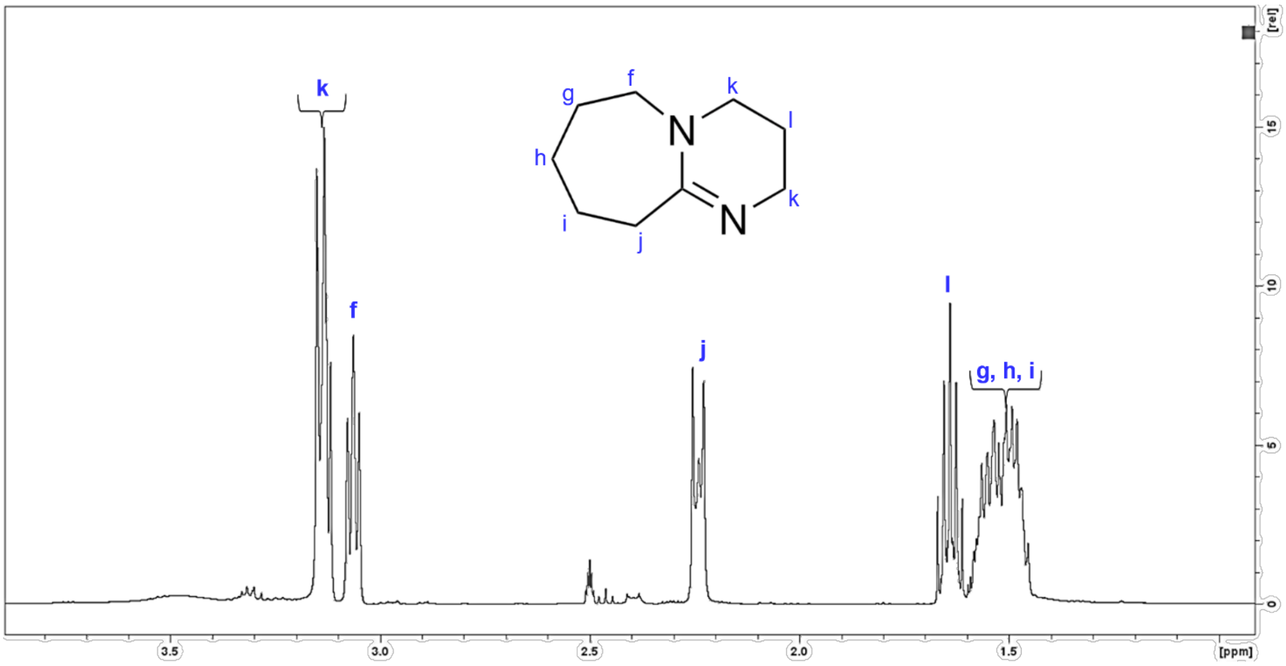

Figure S5. ^{1}H-NMR spectra of DBU. The peak assignment refers to the structures in Table S1.

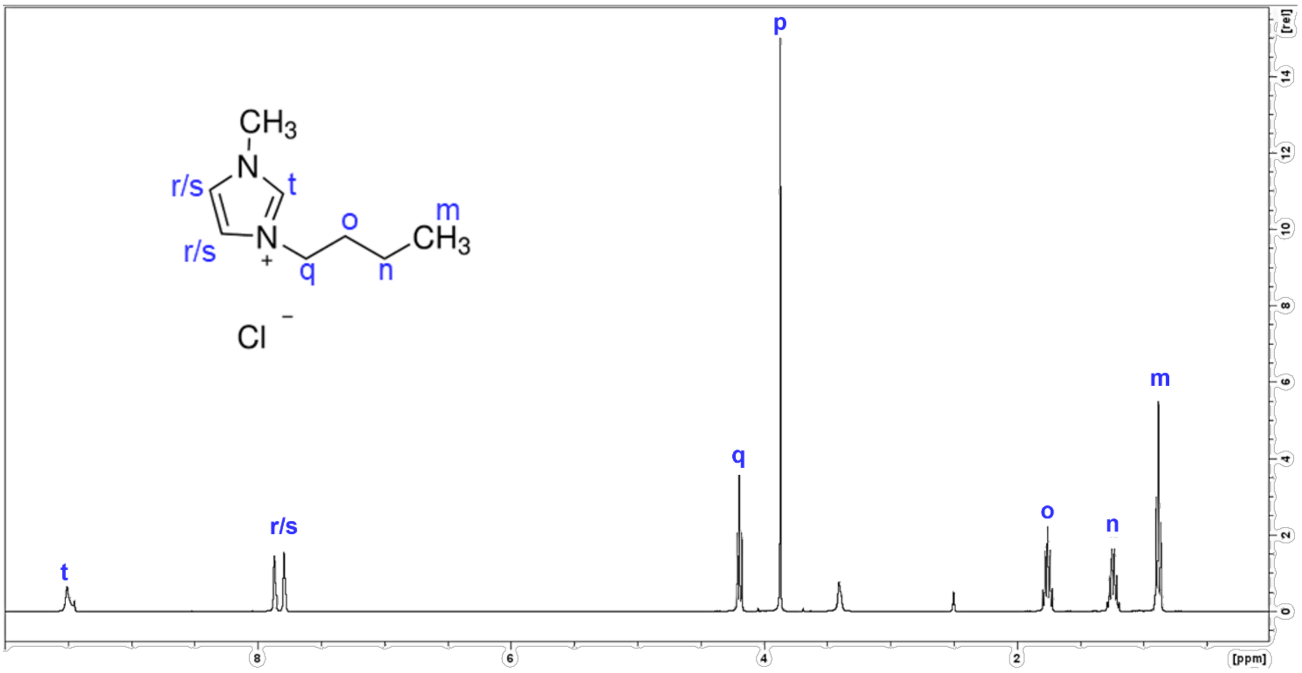

Figure S6. ^{1}H-NMR spectra of [Bmim][Cl]. The peak assignment refers to the structures in Table S1.

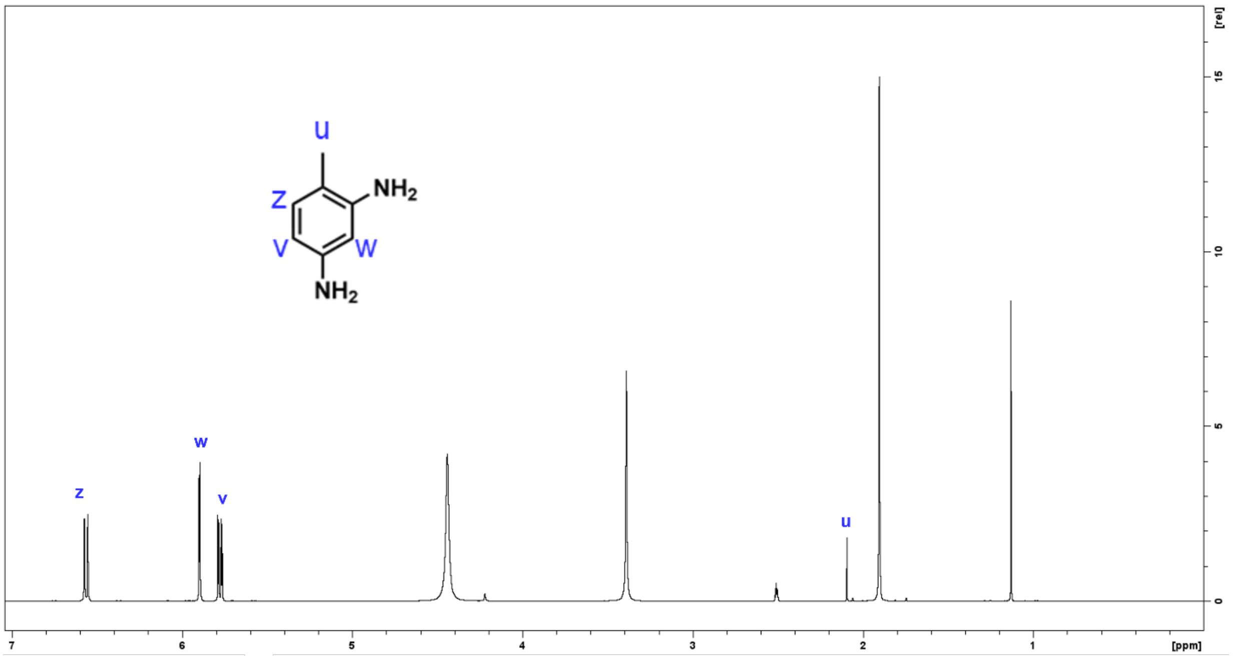

Figure S7. ^{1}H-NMR spectra of 2,4-TDA. The peak assignment refers to the structures in Table S1.

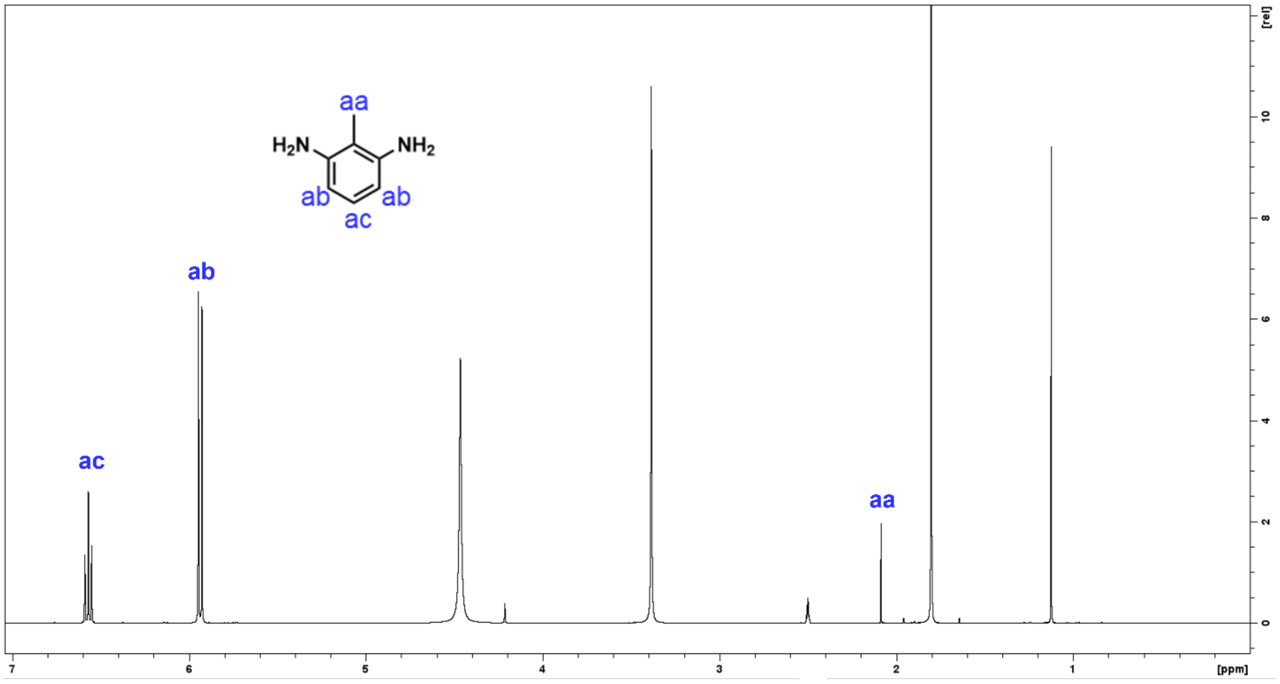

Figure S8. ^{1}H-NMR spectra of 2,6-TDA. The peak assignment refers to the structures in Table S1.

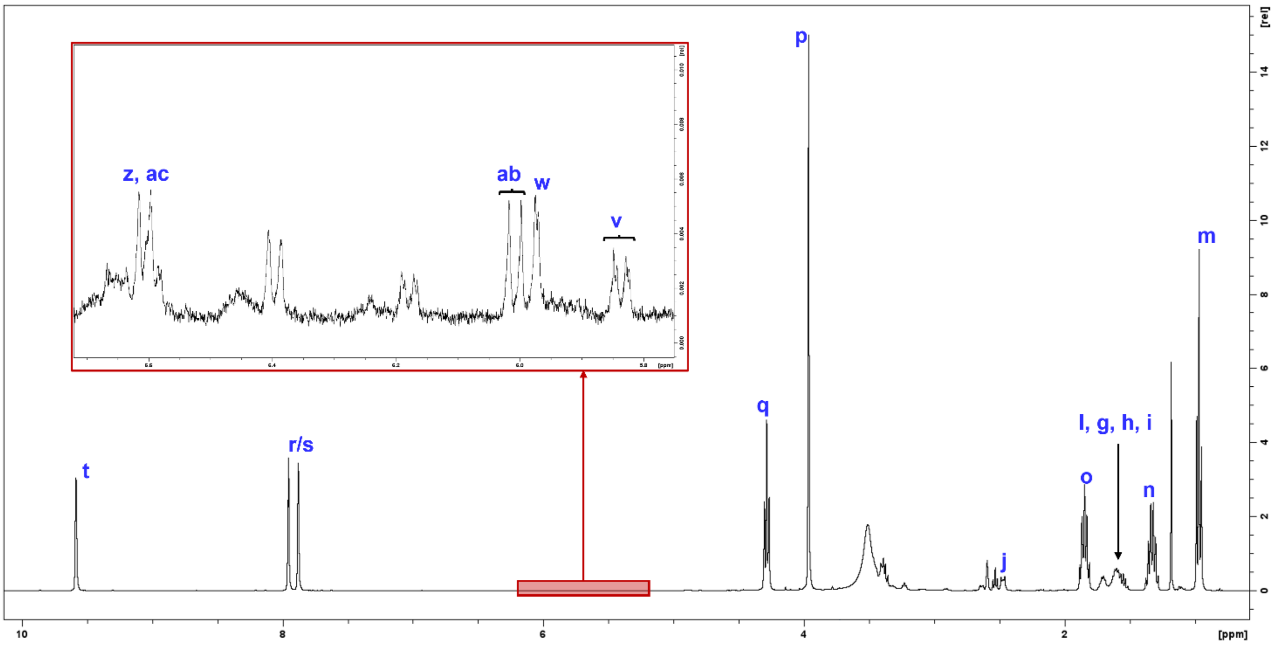

Figure S9. ^{1}H-NMR spectra of the depolymerization medium(DM) after concentration, with a zoomed-in view of the 5.7–6.7 ppm region. The peak assignment refers to the structures in Table S1.

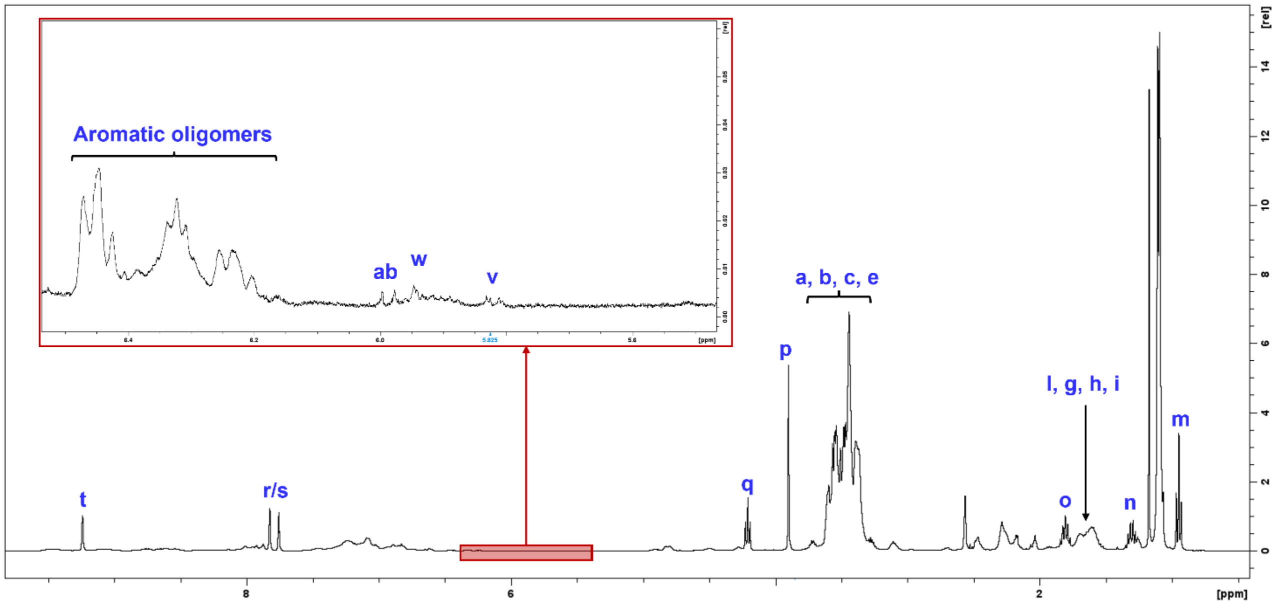

Figure S10. ^{1}H-NMR spectra of the recovered polyol (RP) after concentration, with a zoomed-in view of the 5.7–6.7 ppm region. The peak assignment refers to the structures in Table S1.

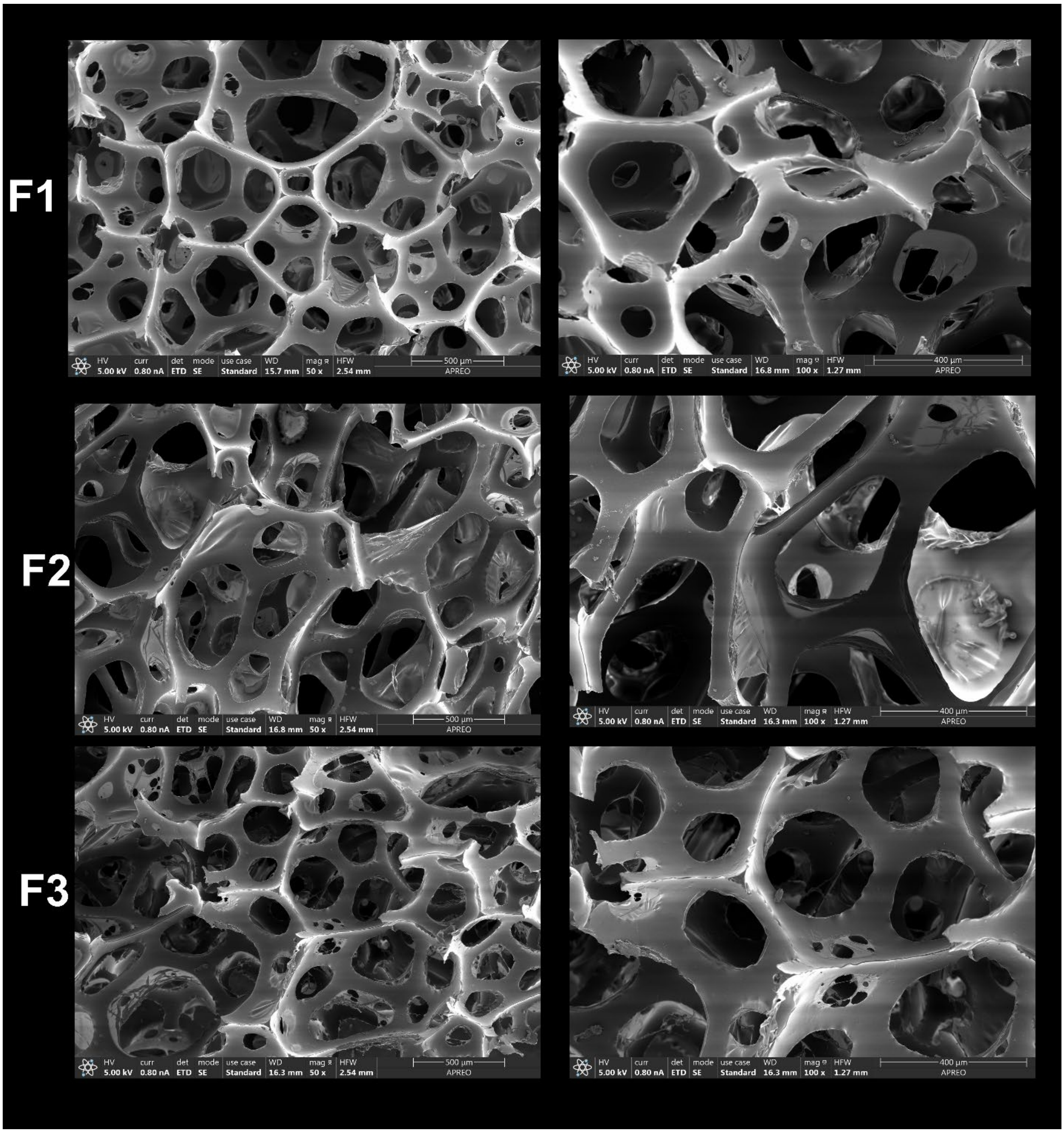

Figure S11. Scanning electron micrographs of cross-sections of the PUFs synthesized using the formulation described in Table 2, shown at two magnifications (50× and 100×). Foam samples include: (F1) reference foam prepared with 100 wt% VP, (F2) foam incorporating 5 wt% of CRP, and (F3) foam containing 5 wt% of the RP.

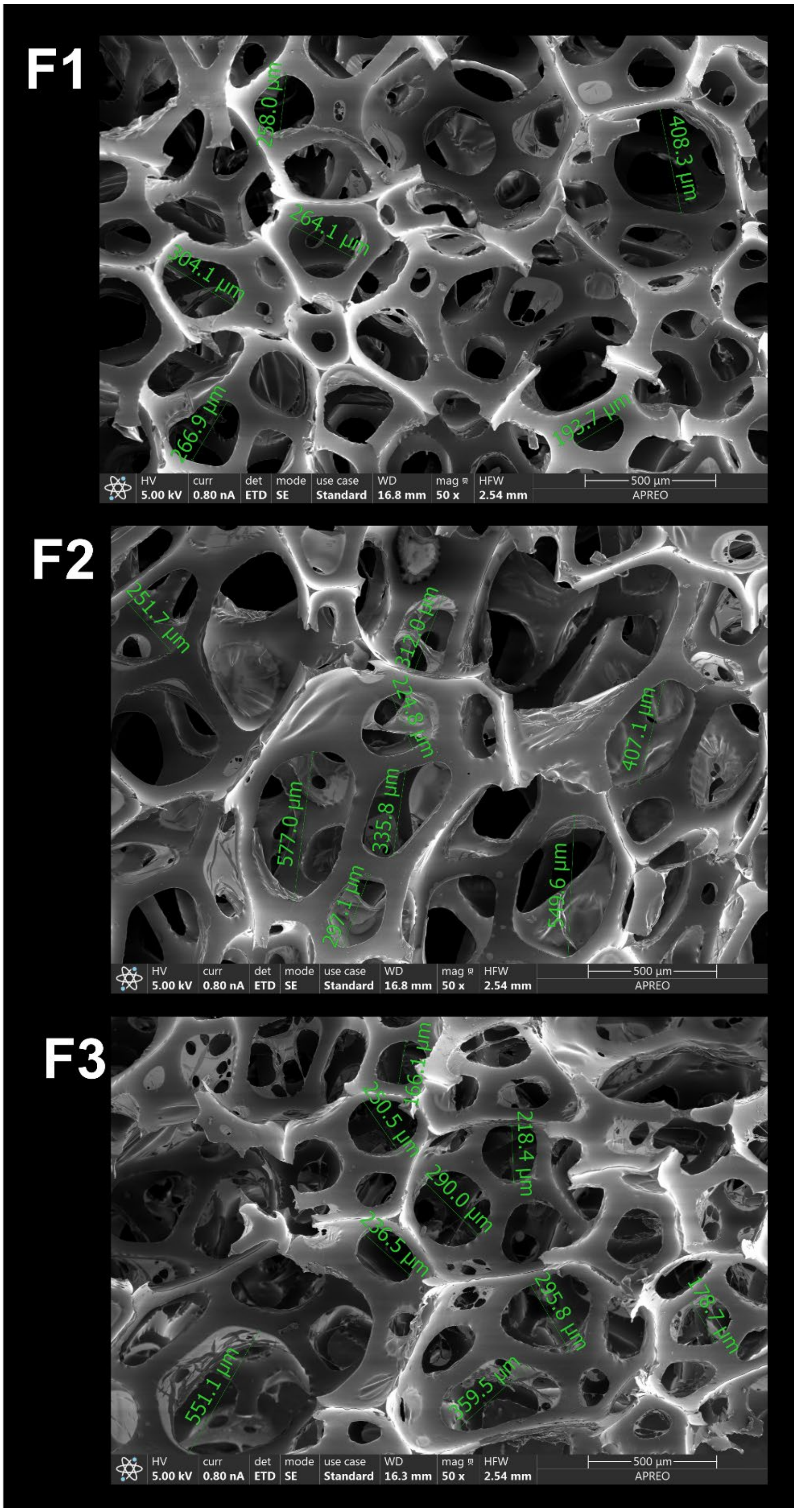

Figure S12. Scanning electron micrographs of cross-sections of the PUFs synthesized using the formulation described in Table 2, with cell diameters indicated within the images. Foam samples include: (F1) reference foam prepared with 100 wt% VP, (F2) foam incorporating 5 wt% of CRP, and (F3) foam containing 5 wt% of the RP.

2. Free aromatic amines quantification

2.1. Quantification of free aromatic amines in the depolymerization medium.

1. Sample preparation.

For the concentrated depolymerization medium, 50 µL of the sample were dissolved in 435 µL of DMSO-δ6 and 15 µL of tert-butanol 1 M in DMSO-δ6.

2. ^{1}H-NMR integration.

Table S2. Monomeric aromatic amines concentration within the depolymerization medium (DM).

Characteristic Chemical Shift(s),ppm[1]	Protons	Area	Area / H	Concentration (mM)
**-CH$_3$ (t-BuOH)[2]: 1.18 ppm**	9	1	0.1	30
**v: 5.77 ppm**	1	0.0033	0.0033	**[Àmines]**
**w: 5.89 ppm**	1	0.0025	0.0025	
**ab: 5.94 ppm**	2	0.0034	0.0017	
**z: 6.56 ppm**<br>**ac: 6.57 ppm**	2	0.0055	0.00275	

[1] The peak assignment refers to the structures in Table S1; [2] Internal standard (*t*-butanol, 1M) used for quantification.

3. Concentration calculation.

$$\left(\frac{Area}{H}\right)_{average} = 0.00256\,;$$

$$[Amines] \quad = \frac{0.00256 \cdot 30}{0.1} = 0.768\, mM$$

2.2. Quantification of free aromatic amines in the recovered polyol.

1. Sample preparation.

Alternatively, for the dry solid product, 40 mg were dissolved in 500 μL of DMSO-δ_6 and then centrifugated. Finally, 485 μL of the supernatant were mixed with 15 μL of tert-butanol 1 M in DMSO-δ_6.

2. ^{1}H-NMR integration.

Table S3. Monomeric aromatic amines concentration within the recycled polyol (RP).

Characteristic Chemical Shift(s),ppm[1]	Protons	Area	Area / H	Concentration (mM)
**-CH_3 (*t*-BuOH)[2]: 1.18 ppm**	9	1	0.1	30
**v: 5.77 ppm**	1	0.0021	0.0021	
**w: 5.89 ppm**	1	0.0016	0.0016	**[Amines]**
**ab: 5.94 ppm**	2	0.0021	0.00105	

The peak assignment refers to the structures in Table S1; [2] Internal standard (*t*-butanol, 1M) used for quantification.

3. Concentration calculation.

$$\left(\frac{Area}{H}\right)_{average} = 0.00158\ ;$$

$$[Amines] \quad = \frac{0.00158 \cdot 30}{0.1} = 0.474\ mM$$
